# RAMBLE ON: LONELINESS AND OFF-TOPIC VERBOSITY IN OLDER ADULTS

**DOI:** 10.1093/geroni/igz038.1952

**Published:** 2019-11-08

**Authors:** Leah N Smith, Jessica H Helphrey, Jennifer Sawyer, Leigh A Fierro, Ben K Mokhtari, Jenna M Moore, Daniel Rodriguez, Michael D Barnett

**Affiliations:** 1 The University of Texas at Tyler, Tyler, Texas, United States; 2 The University of Texas at Tyler, Texas, United States

## Abstract

Numerous studies have found off-topic verbosity occurs more frequently in older adults than younger adults. Previous theories have attributed this to age-related decline, emotion recognition, and communication style. Previous research has linked lower loneliness with more off-topic verbosity; however, the precise nature of this relationship remains unclear. Loneliness has been defined as an inconsistency between an individual’s actual and desired social relationships, and previous research has found that loneliness is associated with lower cognitive and social outcomes among diverse populations including older adults. The purpose of this study was to investigate the relationship between loneliness and off-topic verbosity among older adults. Healthy, community dwelling older adult participants (N = 82; age 60-99, M = 76.66, SD = 8.52) completed the Three-Item Loneliness Scale and provided a verbal sample in which they recounted an autobiographical memory (a vacation) and a procedural memory (how to make a breakfast); the verbal samples were transcribed and rated by three independent judges. In contrast with previous research, results found that loneliness was associated with a greater tendency to engage in tangential verbal topics. This suggests that social factors such as loneliness may impact the way some older adults express themselves verbally.

